# The Role of Transurethral BPH Surgeries in Management of Urinary Symptoms in Prostate Cancer Patients, Narrative Review

**DOI:** 10.1007/s11934-024-01229-1

**Published:** 2024-10-01

**Authors:** Mohamed Elsaqa, Marawan M. El Tayeb

**Affiliations:** 1 Alexandria University Faculty of Medicine, Sultan Hussein Street, Alazarita, Alexandria, 21131 Egypt; 2 Division of Urology, Department of Surgery, Baylor Scott & White Health, Temple, TX USA

**Keywords:** Lower urinary tract symptoms, BPH, Prostate cancer, TURP, HoLEP, PVP

## Abstract

**Purpose of Review:**

Prostate cancer and benign prostate hyperplasia (BPH) are two ubiquitous pathologies that may coexist. A significant percentage of patients with different stages of prostate cancer suffer lower urinary tract symptoms (LUTS) due to associated BPH. We aimed to review the literature regarding the role of transurethral surgeries in the management of prostate cancer patients and the different available management options.

**Recent Findings:**

The evidence in literature for the use of BPH surgeries in prostate cancer patients is based mainly on low-quality retrospective studies. In patients on active surveillance, BPH surgeries are beneficial in relieving LUTS without oncological risk and can eliminate the contribution of adenoma to PSA level. In patients with advanced prostate cancer, palliative BPH surgery can relieve LUTS and urinary retention with unclear oncological impact; however some reports depict that the need for BPH surgery in advanced prostate cancer is associated with poorer prognosis. In patients receiving radiotherapy, various studies showed that transurethral resection of prostate (TURP) is associated with increased radiotoxicity despite some recent reports encouraging the use of Holmium Laser Enucleation of the Prostate (HoLEP) to improve urinary symptom scores before radiotherapy. The most commonly reported techniques utilized are TURP, photoselective vaporization of prostate (PVP) and HoLEP.

**Summary:**

The use of BPH surgery is justified for relieving LUTS in selected prostate cancer patients on active surveillance or in advanced stages, however the use in the pre-radiotherapy settings remains controversial. Future prospective and randomized controlled trials are required for validating the benefits and assessing potential hazards.

## Introduction

Prostate cancer is the most common cancer and the second leading cause of mortality among men in the United States. In 2024, an estimated 299,010 U.S. men will be diagnosed with prostate cancer with estimated resultant 35,250 deaths [1, 2]. Most prostate cancer patients are nowadays diagnosed at an early stage [3]. According to Maganty et al., radiation therapy is the most common treatment in patients with newly diagnosed prostate cancer and represents 47% of patients compared to active surveillance (AS) (23.4%), surgery (20.6%) and hormonal therapy (7.9%) [4]. 

Benign prostatic hyperplasia (BPH) may coexist in prostate cancer patients and cause significant lower urinary tract symptoms (LUTS). In patients on AS for prostate cancer, about 34% and 6% of patients suffer moderate and severe LUTS respectively [5]. Moreover, radiotherapy for localized prostate cancer is usually associated with a surge in LUTS especially in patients with significant baseline symptom scores [6]. Patients with locally advanced or metastatic prostate cancer may also suffer LUTS or urinary retention either due to associated adenoma or tumor growth [7, 8]. 

We aimed to review the literature regarding the role and options of BPH transurethral procedures in management of urinary symptoms in prostate cancer patients of different categories.

## Evidence Acquisition

A comprehensive review of literature was performed for the data relating to use of BPH surgeries for prostate cancer patients. Relevant information has been gathered, collated and reviewed to deliver a practical summary for clinicians. Bibliographic databases searched included PubMed, Web of Science, Google Scholar and Cochrane Library. The use of transurethral procedures was discussed in patients of the following stages of prostate cancer: on AS, receiving radiotherapy, and patients with advanced or metastatic disease.

### Patients on Active Surveillance (AS) for Prostate Cancer

Some studies have reported the use of transurethral procedures for managing obstructive symptoms in patients on AS for prostate cancer. All the reported studies were of retrospective nature. All the studies reported the safety of transurethral procedures in those patients with no added morbidity and no impact on the disease progression rates. As the most common procedure utilized for BPH, TURP is also the most reported procedure used in this purpose followed by PVP and HoLEP. No studies have compared the outcome of different techniques in this specific purpose.

- **TURP**: Koo et al. reported the use of TURP in 86 patients on AS patients with median prostate volume of 45 gm. With median follow up of about 50 months, 21 (24.4%) patients remained on AS [9]. Hagmann et al. have recently reported the long-term follow-up data on the use of TURP in 413 men with median follow up of 46 months. Cancer specific survival was 99.7% and overall survival was 92.3%. Half of patients on AS eventually required definitive treatment due to disease progression, however half of patients have benefited from delaying active treatment and are spared the negative side effects of active treatment [10].
- **PVP**: PVP was also reported for the use in patients on AS by Jibara et al. Their cohort of 71 patients had substantial improvement of urinary symptoms with low complication rate. At 3 years of follow up, the probability of remaining on surveillance was 93% [11].
- **HoLEP**: Schober et al. reported the use of HoLEP for management of refractory LUTS in a small cohort of 20 patients on AS with significant improvement in voiding parameters [12]. Our institution reported HoLEP use in 117 localized prostate cancer patients. With median follow up of 30 months, 88 (72%) patients remained on AS. The complication rate was low and comparable to non-surgical BPH patients [13].
- **Predictive value of PSA following transurethral procedures in active surveillance (AS) patients**: Benign prostate adenoma contributes to serum PSA in prostate cancer patients. With larger adenoma, higher serum PSA level is seen. High PSA levels in AS patients may trigger prostate biopsy or proceeding to definitive treatment. Studies reported reduction of serum PSA following transurethral procedures in patients on AS for low-risk prostate cancer. This reduction of PSA through reducing the contribution of large adenoma to PSA level can help minimize PSA anxiety, give better predictive value for PSA level and reduce the need for prostate biopsies.

In a large series of more than 800 patients, Wu et al. showed that levels of PSA following TURP might be helpful for risk stratification and the selection of patients for conservative management. They reported that greater PSA reduction following TURP, and post-TURP PSA ≤ 4 were associated with better progression-free survival (PFS). They also reported that local therapy improved PFS in intermediate- and high-risk groups, but not in the low-risk group. Regardless of pre-TURP PSA, no improvement in PFS was found with post-TURP PSA level ≤ 4 ng/ml [14]. Koo et al. reported that TURP reduced the PSA by 34.5% and the PSA density by 50.0%. This had converted 58 out of 71 (81.7%) patients ineligible for AS based on PSA level and density to eligible features [9].

This was demonstrated with PVP by Jibara et al. At 12 months post-PVP, PSA levels decreased by 36% [11]. Moreover, Schober et al. showed a drop of median PSA following HoLEP from 8.5 ng/mL to 1.4 ng/mL at median follow-up of 18.5 months [12]. In our reported HoLEP cohort, median PSA has decreased from 7.6 ng/ml before HoLEP to 1.3, 1.4, and 1.7 ng/mL at 6-week, 3-month, and 1-year follow-up respectively [13].

### Patients Receiving Radiotherapy for Localized Prostate Cancer

#### The Morbidity of Radiation Therapy in Prostate Cancer

External beam radiation therapy for prostate cancer is associated with risk of urinary and gastrointestinal toxicity. Up to 80% of patients may suffer moderate to severe manifestations of urinary toxicity. Urinary toxicity is usually classified as acute and late toxicity. Acute toxicity occurs during and within 3–6 months after treatment, while late toxicity starts 6 months after treatment and often may start many years after treatment [15, 16].

Urinary toxicity manifests as frequency, urgency, dysuria, hematuria, or urinary incontinence. The pathophysiology of urinary radiation injury is still not completely understood. Radiation damages the urothelium, detrusor muscles and blood vessels resulting in acute inflammation that finally leads to reduced bladder capacity, hypocompliance, reduced urinary functionality and possible urethral stricture [16].

Compared to 3D-Conformal radiotherapy (CRT), intensity modulated radiotherapy (IMRT) significantly reduced the risk of acute and late toxicity of grade 2 or higher. Recent advances have also encouraged dose escalation with hypofractionated schedules with considerable reduction of toxicity. However, local urinary and gastrointestinal toxicity still remains a limiting factor [17].

#### Predictors of Urinary Toxicity in Prostate Cancer Radiotherapy

Multiple studies have evaluated the predictors of urinary toxicity associated with pelvic radiotherapy. A first essential risk factor for urinary toxicity, consistently described by different studies, is the baseline urinary functionality—with patients having baseline impaired functionality and high International Prostate Symptom Score (IPSS) being at higher risk of experiencing severe acute and late urinary toxicity [15, 18–23]. Pisani et al. have reported that baseline IPSS value and presence of nocturia and urinary incontinence at baseline assessment were associated with acute toxicity [18]. 

Rancati et al. have shown that factors associated with risk of urinary toxicity include baseline urinary symptoms, previous TURP, smoking, age, vascular comorbidities, and use of cardiovascular drugs [15]. According to Yahya et al., baseline symptoms, age, and diabetes mellitus were the main predictors of late toxicity in multiple follow up intervals. Other less strongly associated factors include cerebrovascular condition, ECOG status and non-steroidal anti-inflammatory drug usage [19]. Natesan et al. studied the influence of prostate volume on the toxicity of prostate radiation. They showed that large initial prostate volume and higher baseline IPSS score were associated with increased risk and earlier onset of urinary toxicity [24].

#### Urinary Symptoms Optimization Prior to Radiotherapy

Accordingly, an evaluation of the baseline urinary symptoms should be mandatory before planning for radiotherapy. Assessment should include IPSS and may require cystoscopy or urodynamics [16]. Martin et al. recommended that pre-emptive TURP should be considered in patients with moderate to high IPSS and those with clinically substantial obstructive urodynamics [16]. One recent study by Laughlin et al. have reported the outcome of EBRT in patients with prior history of HoLEP in a small cohort of 18 patients. They reported low risk of early or late complications. They recommended the use of HoLEP in patients with significant bladder outlet obstruction prior to EBRT [25]. We reported similar findings in our published cohort of 27 patients [13]. 

Prostate volume reduction prior to radiotherapy or brachytherapy in the aim of reducing urinary toxicity through androgen deprivation therapy (ADT) or antiandrogens was proposed in some studies [26–29]. Initial large prostate volume seems to be the highest predictor for prostate volume reduction by hormonal therapy [29]. Combined androgen blockade was superior to antiandrogen monotherapy in reducing prostate volume according to Majumder et al., however, was not reflected as a difference in quality of life during radiotherapy or brachytherapy [27]. Langenhuijsen et al. showed that the maximal volume reduction before prostate radiotherapy by neoadjuvant ADT is achieved at 6 months while in small prostates 3 months may be sufficient [26]. No comparative studies has been conducted to prove the impact of neoadjuvant hormonal therapy on improving the urinary symptoms or reducing the toxicity during radiotherapy.

#### Urinary Toxicity in Radiotherapy Patients with History of Prior TURP

Multiple studies have reported increased urinary toxicity in patients with a history of TURP prior to prostate radiotherapy. All the studies are of retrospective nature and mostly of small number of patients. The findings of the related studies are summarized in Table 1 [30–36]. In their recent review article of EBRT toxicity with history of TURP, Neerhut et al. showed that history of TURP was not associated with increased risk of acute toxicity, but increased risk of late toxicity— particularly hematuria. The factors linked to increased toxicity were large prostatic resection cavity, receiving radiotherapy within 6–12 months of TURP, high baseline IPSS score (> 19), large prostate volume (> 40 cc), more than one TURP and high dose radiotherapy to prostate cavity [37].

**Table 1:** Summary of the studies assessing the impact of TURP on prostate cancer radiotoxicity

Study	Year	No. of patients	Type of radiotherapy	Findings
Sandhu et al. [30]	2000	120	EBRT (3D-CRT)	- Higher risk of stress incontinence is observed compared to patients without prior TURP. - The incidence of high-grade urinary toxicity was low.
Devisetty et al. [31]	2009	71	EBRT	- Prior TURP was associated with higher rate of acute or late Grade ≥ 3 toxicity. - Acute toxicity was increased in patients with a history of more than 1 TURP. however, the overall incidence of GU toxicity is low, and toxicity tends not to persist.
Lee et al. [32]	2016	96	EBRT (proton therapy)	-High incidence of toxicity and inferior QOL scores in patients with history of TURP.
Molineros et al. [33]	2021	34	EBRT	- Significantly more secondary surgeries and significantly more additional interventions compared to patients with TURP for BPH. - Compared to patients who had TURP after EBRT, no difference in complication rate or oncological outcome.
Merrick et al. [34]	2004	27	Low dose Brachytherapy	- Patients with pre-implant TURP had comparable urinary QOL to that of patients without pre-implant TURP.
Peddada et al. [35]	2007	28	High dose brachytherapy	A single arm study showed low morbidity in patients with prior TURP.
Luo et al. [36]	2009	32	High dose Brachytherapy	- Genitourinary toxicity was comparable between patients with or without previous TURP.

### Patients with Advanced/Metastatic Prostate Cancer

- **TURP**: Few studies have reported the use of palliative TURP in patients with advanced prostate cancer. The procedure is usually known as “Channel or Tunnel TURP”. All the studies are of retrospective cohort nature. Studies reported the benefit of TURP for reliving obstruction and improving QOL with low morbidity in patients with advanced prostate cancer [38–44]. Compared to patients undergoing TURP for BPH, few studies reported higher rates of postoperative urinary retention and reoperation, especially in patients with hormone refractory disease [7, 44]. Another study showed a higher rate of urinary incontinence [45].
- **PVP**: PVP use for palliative management of advanced prostate cancer has been reported in a few small volume cohort studies [46–49]. The studies reported the safety and efficacy of PVP in improving urinary symptoms or relieving urinary retention in those patients with minimal morbidity or complications.
- **PVP vs. TURP**: Altay et al. compared TURP and PVP in management of 74 patients with advanced prostate cancer. They showed that both techniques achieved comparable IPSS scores, however PVP had the advantage of earlier catheter removal, shorter hospital stay and lower incidence of urethral stricture [50].

#### The Impact of BPH Procedures on Oncological Outcome in Patients with Advanced Disease

The need for TURP in patients with advanced prostate cancer was an adverse prognostic factor and associated with worse outcomes in some studies [38–43]. Some older studies have compared the outcomes of prostate cancer patients diagnosed through needle biopsy and those diagnosed through TURP. Those studies reported that TURP was associated with worse disease-free survival, especially in patients with T3 and T4 disease and tumors of intermediate to poor differentiation [38, 51, 52]. On the contrary, Qu et al. and Chen et al. showed that TURP and PVP respectively were associated with oncological benefit and better cancer specific survival in patients with metastatic prostate cancer compared to receiving ADT alone [8, 47].

- A recent database-dependent report by Marchioni et al. presented the surgical outcomes of transurethral surgeries of any type in prostate cancer patients, collectively of any stage, in comparison to the outcome in patients with BPH. In their report, TURP was the most common technique (78.4%) followed by PVP (11.5%) and Enucleation (10.1%). The postoperative complications, blood transfusions, length of hospital stay and perioperative mortality in patients of prostate cancer were statistically comparable to those with BPH [53].

## Conclusions

- Although the need for transurethral surgeries in patients with prostate cancer is not rare, the level of evidence is weak and is based on retrospective cohort studies of usually a small number of patients.
- The use of transurethral surgeries in patients on AS seems safe and effective with no negative oncological impact. PSA drop after transurethral surgery in those patients may help eliminate PSA anxiety and need for prostate biopsies.
- Transurethral surgeries in the pre-radiation settings are associated with increased radiation toxicity. The use of hormonal therapy appears beneficial for decreasing prostate size and improving LUTS.
- In patients with advanced prostate cancer, transurethral surgeries are safe and effective for palliation of LUTS and improving QOL. However, the need for transurethral surgeries may be linked to worse prognosis.
- The most common BPH procedure used is TURP. PVP and HoLEP have been proposed as alternatives to lower morbidity, however, none of these techniques has proven superior to the others.
- Future randomized trials and well-controlled prospective studies with larger patient populations are required to better validate the role of transurethral surgeries in prostate cancer patients.

## Key References

- Hagmann S, Ramakrishnan V, Tamalunas A, Hofmann M, Vandenhirtz M, Vollmer S, et al. Two Decades of Active Surveillance for Prostate Cancer in a Single-Center Cohort: Favorable Outcomes after Transurethral Resection of the Prostate. Cancers (Basel). 2022 Jan 12;14(2):368.
  - Outcome of TURP in patients on active surveillance for prostate cancer.
- Natesan D, Carpenter DJ, Floyd W, Oyekunle T, Niedzwiecki D, Waters L, et al. Effect of Large Prostate Volume on Efficacy and Toxicity of Moderately Hypofractionated Radiation Therapy in Patients With Prostate Cancer. Adv Radiat Oncol. 2022 Mar;7(2):100805.
  - Impact of prostate volume and baseline urinary symptoms on the radiation toxicity for prostate cancer.
- Laughlin BS, Narang GL, Cheney SM, Humphreys MR, Vargas CE, Keole SR, et al. Toxicity and outcomes after external beam irradiation for prostate cancer in patients with prior holmium laser enucleation of the prostate: Early experience. Cancer Rep. 2023 Jan 5;6(1).
  - A recent report of the benefit of prostate volume reduction prior to radiotherapy using HoLEP.
- Neerhut T, Grills R, Lynch R, Pr1ece PD, McLeod K. Genitourinary toxicity in patients receiving TURP prior to hypofractionated radiotherapy for clinically localized prostate cancer: A scoping review. Urologic Oncology: Seminars and Original Investigations. 2024 Jun;42(6):165–74.
  - A recent review article of the radiation toxicity in patients with prior TURP.
- Marchioni M, Primiceri G, Veccia A, Di Nicola M, Carbonara U, Crocerossa F, et al. Transurethral prostate surgery in prostate cancer patients: A population-based comparative analysis of complication and mortality rates. Asian J Urol. 2024 Jan;11(1):48–54.
  - A recent report of the outcome of transurethral surgeries in patients with prostate cancer compared to BPH patients.

 

## Data Availability

No datasets were generated or analysed during the current study.
